# Interleukin 4, interleukin 6 and osteopontin-serological markers of head and neck malignancy in primary diagnostics: A pilot study

**DOI:** 10.3892/ol.2014.2312

**Published:** 2014-07-04

**Authors:** CHRISTOPH ADERHOLD, GUIDO MANUEL GROBSCHMIDT, ALEXANDER SAUTER, ANNE FABER, KARL HÖRMANN, JOHANNES DAVID SCHULTZ

**Affiliations:** Department of Otorhinolaryngology, Head and Neck Surgery, Medical Faculty of Mannheim, University of Heidelberg, Mannheim D-68167, Germany

**Keywords:** vascular endothelial growth factor, granulocyte-colony stimulating factor, platelet-derived growth factor, osteopontin, epidermal growth factor receptor, interleukin-4, serological markers, interleukin-6, head and neck squamous cell carcinoma

## Abstract

The progression of head and neck squamous cell carcinoma (HNSCC) is stimulated by various angiogenic peptides and growth factors. A correlation between tumor progression and the secretion of various serological mediators in patients with malignant tumors of the head and neck is of major interest for tumor diagnostics, evaluation of the therapy response and it may predict prognosis by specifying the individual tumor biology. Established chemotherapeutic regimes for head and neck tumors usually consist of platinum-based chemotherapeutic drugs and 5-fluorouracil (5-FU). The present pilot study sought to assess the eligibility of seven serological factors as biomarkers for malignant tumors of the head and neck: Platelet-derived growth factor, vascular endothelial growth factor, epidermal growth factor receptor, osteopontin, granulocyte-colony stimulating factor, interleukin-4 (IL-4) and IL-6. The serum levels of each factor in 20 patients receiving concomitant radiochemotherapy with cisplatin or carboplatin and 5-FU with curative intent were determined prior and subsequent to chemotherapy and were compared with 40 healthy controls. Another aim of the pilot study was to investigate whether the serum of patients showed significant differences in the concentrations of the analyzed factors at the start of concomitant radiochemotherapy compared with the controls, whether those markers indicated a neoplastic process and whether concomitant radiochemotherapy with cisplatin or carboplatin and 5-FU induced significant alterations of concentration compared with pre-therapeutic levels. The included patients were histopathologically diagnosed with HNSCC and the average age was 62.3 years. The serum samples of the patients were obtained during the course of regular pre- and post-chemotherapeutic blood draws one week prior to the start of radiochemotherapy and one week following the completion of chemotherapy. The healthy controls were collected from patients of the Sleep Laboratory of the Department of Otorhinolaryngology, Head and Neck Surgery, University Hospital (Mannheim, Germany) without clinical evidence or laboratory signs of inflammation or history of a malignant disease. The average age was 50.3 years. The serological level of each factor was ascertained by enzyme-linked immunosorbent assay in duplicate. Serum levels of IL-4, IL-6 and osteopontin were significantly increased in patients with HNSCC compared with those in chemotherapy-naive healthy controls. IL-4 and osteopontin showed no significant therapy-associated alterations. Notably, IL-6 levels significantly increased post-therapeutically. Using logistic regression with osteopontin and IL-4, an individual risk-profile for random samples was calculated. IL-4, IL-6 and osteopontin appear to be suitable indicators of the neoplastic process as they are significantly increased in HNSCC patients compared with the control group. With the exception of IL-6, whose levels were in fact increased following therapy, a significant therapy-associated alteration of these factors was missing. Therefore, these serological markers failed to predict the therapy response, but they may be valuable as a screening instrument in primary diagnostics.

## Introduction

Tumors of the head and neck form a heterogeneous group of malignant neoplasms that typically arise from the upper aerodigestive tract. The most common tumor entity (>90%) is head and neck squamous cell carcinoma (HNSCC). HNSCC commonly affects the oral cavity, the hypo-and oropharynx and the larynx. HNSCC customarily originates from epithelial layers and often from pre-cancerous lesions, including leukoplakia. Histologically, HNSCCs are subclassified as verrucous, basaloid and adenosquamous carcinomas. In 2008, the World Health Organization calculated ~631,800 new cases of HNSCC globally. This equates to a global incidence of 13.7/100,000 (1). HNSCC is the result of a multifactorial process caused by carcinogenic substances (2–4). Chronic consumption of alcohol and tobacco abuse are the main risk factors for HNSCC. Between 85 and 90% of all HNSCC cases are associated with nicotine or alcohol abuse (2). In addition, the risk for HNSCC rises with the amount and duration of abuse. Consequently, a synergistic effect induced by alcohol and nicotine has been hypothesized (5). HNSCCs have a high invasive potency and even an early-stage tumor is at risk for lymphogenic metastasis. In this context, the topographical affection is linked to the cervical lymph nodes (6). Subsequent to incorporation into the subcapsular sinus of the lymph nodes, the tumor cells start to proliferate (7). Tumor size and location, lymph node invasion, extracapsular spread and distant metastases define the individual tumor prognosis without taking account of the heterogeneous tumor biology of the tumor entity.

Vascular endothelial growth factor (VEGF) is a highly potent angiogenic factor that is strongly expressed in a multitude of neoplasias, including breast, lung and head and neck cancer (8). It has been shown that the serological VEGF levels of patients with head and neck cancer correlate with the occurrence of lymph node metastasis and a poor prognosis (9). Furthermore, high levels of other angiogenic factors, for instance platelet-derived growth factor (PDGF), and a high rate of p53 mutations have been reported for cases of HNSCC with elevated VEGF levels (10,11).

Various isoforms of PDGF are involved in inflammatory and angiogenic processes and in cellular migration of HNSCC. The autocrine stimulation caused by PDGF leads to tumor growth and facilitates the infiltration of tumor stromal cells (12). In contrast to healthy controls, patients with HNSCC show significantly higher PDGF levels, but there is no significant correlation between clinical stage and the PDGF serum level (13).

The epidermal growth factor receptor (EGFR) is a tyrosine kinase that is expressed in normal tissue and in tumor cells. When EGFR is activated by its physiological ligands, transforming growth factor-α or EGF, various enabled G-protein linked kinases affect the transcription and secretion of growth-enhancing mediators. These mediators lead to autocrine and paracrine stimulation of pathological growth and the angiogenic affiliation of tumor cells in head and neck cancer (14).

Interleukin-4 (IL-4) is an anti-inflammatory cytokine that is produced and secreted by type 2 T-helper cells and mast cells, and that plays a crucial role in allergic reactions of the skin and mucosa membrane. It has been shown that SCCs do not produce IL-4 (15), but IL-4 expression can be found in the tumor stroma (16). *In vitro* studies have shown that IL-4 triggers tumor growth of HNSCC cell cultures in a dose-dependent manner (16,17). By contrast, a growth-inhibiting effect was reported for melanomas and gastric and renal cancer (18–20). Additionally, IL-4 has shown an antiangiogenic effect in animal studies (15,21).

As a multifunctional cytokine, IL-6 has proinflammatory properties and activates migration of immune cells. Increased IL-6 levels have been determined in lung, ovarian and head and neck cancer (22,23). Wang *et al* (24) showed that patients suffering from malignant tumors of the head and neck exhibited distinctly elevated IL-6 and IL-6-receptor levels compared with healthy controls. The occurrence of metastases, relapses and reduced overall survival rates were also significantly associated with elevated serological IL-6 levels (24).

Osteopontin is an extracellular phosphoglycoprotein that is physiologically involved in the formation of bone matrix. With its ability to mediate cell adhesion, osteopontin can take part in the process of tumor invasion, angiogenesis and metastasis formation. Elevated osteopontin levels have been reported for 34 various tumor entities and their metastasis (25). Weber *et al* (26) revealed that low osteopontin levels prior to therapy were associated with higher overall survival rates and an improved therapy response in patients with head and neck cancer. By contrast, Lim *et al* (27) failed to verify a correlation between osteopontin and the overall survival rate or therapy response in head and neck cancer. However, osteopontin was suitable for use as a tumor marker, although it was not clear for which entity it was most appropriate (26,28).

Granulocyte-colony stimulating factor (G-CSF) is produced and released by macrophages, fibroblasts and epithelial cells that are also part of the tumor stroma (29). In bone marrow, G-CSF works as a mediator to stimulate cell differentiation of the progenitor cells of neutrophil granulocytes. Increased G-CSF levels are detectable in leukemia and also in solid tumors (30). In HNSCC, G-CSF stimulates the proliferation and migration of tumor and inflammatory cells. In contrast to G-CSF-negative tumors, G-CSF-positive tumors show distinct invasiveness of bone and cartilage (31).

The present pilot study assessed the applicability of these seven serological factors as biomarkers for malignant tumors of the head and neck. The pre- and post-therapeutical serum samples were determined from 20 patients receiving concomitant radiochemotherapy with two cycles of cisplatin or carboplatin and 5-fluorouracil (5-FU) with curative intent, and the expression of these markers was compared with that in healthy controls. The pilot study sought to investigate whether the serum of patients showed significant concentration differences in the analyzed factors at the start of concomitant radiochemotherapy compared with the controls, and whether these markers indicated a neoplastic process. The study also examined whether concomitant radiochemotherapy with cisplatin or carboplatin and 5-FU induced significant alterations of concentration compared with pre-therapeutic levels.

## Patients and methods

### Patient characteristics and treatment

The present study was approved by the Ethics Committee II of the Medical Faculty of Mannheim at the University of Heidelberg (file number 2011-279N-MA; Mannheim, Germany). Written informed consent was obtained from all patients and members of the control group. The study assessed 20 patients (17 male and 3 female; mean age, 62.4 years; and range, 41–77 years) and 40 healthy control subjects (25 male and 15 female; mean age, 50.3 years; and range, 19–81 years). All patients underwent concomitant radiochemotherapy due to a malignant tumor of the head and neck with two cycles of 5-FU (1,000 mg/m^2^; treatment days 1–4 and 22–25) and cisplatin (80 mg/m^2^; treatment days 1 and 22) or carboplatin [dose calculated using the Calvert formula (32); treatment days 1 and 22]. All patients were treated with curative intent. No participant received palliative therapy or ‘best supportive care’. A total of 80% of the tumor patients received adjuvant (postoperative) concomitant radiochemotherapy with 60–66 Gy of the tumor localization and 44–66 Gy of the un- or involved nodal levels following surgical resection of the tumor and reconstruction, and uni- or bilateral neck dissection. The remaining 20% of patients with HNSCC underwent definitive radiochemotherapy with two cycles of chemotherapy and a cumulative dose of radiation from 66–74 Gy (primary tumor localization) and 44–64 Gy (un- and involved nodal stations).

In total, 30% of the diagnosed tumors were localized in the oropharynx, particularly in the tonsil area, with 20% in the oral cavity, 20% in the larynx, 15% in the salivary gland, 15% in the lower lip, 5% in the hypopharynx and 15% were cancer of unknown primary syndrome. A total of 10% of the head and neck malignancies were locoregional metastasis (lymph node metastasis) or local tumor recurrences following initial tumor resection. At the initiation of therapy, none of the patients presented with distant metastasis, although 80% of the patients were affected by lymph node metastasis. According to the Union for International Cancer Control classification (33), 75% of the patients had stage IVA cancer, 15% had stage III, 5% had stage I and 5% had stage II. Along with cardiopulmonary comorbidities (arterial hypertension, coronary heart disease and cardiac arrhythmias), diabetes and ethyltoxic hepatic cirrhosis were coexisting. Of the 20 patients, 13 stated regular nicotine use. All patients with HNSCC completed the definitive or postoperative radiochemotherapy. No patients dropped out of the study or had to be excluded. All of the 40 controls were healthy patients from the Sleep Laboratory of the Ear Nose and Throat Department without clinical or laboratory signs of inflammation or a history of a malignancy. During the course of regular pre- and post-chemotherapeutic blood draws, one ethylenediaminetetraacetic acid and one serum sample with S-Monovette^®^ (Sarstedt, Nuembrecht, Germany) was obtained from each patient one week prior to and one week following chemotherapeutic treatment. The interval between pre- and post-therapeutic blood draws was approximately six weeks. The collected samples were centrifuged with 2,000 × g for 10 min and the supernatant plasma was pipetted into Eppendorf^®^ tubes, labeled and stored at −20°C.

### Assays

The serological levels of each factor were measured by enzyme linked immunosorbent assay (ELISA) (R&D Systems, Abingdon, UK). All required reagents were warmed from a storage temperature of 2°C to room temperature for analysis. To prepare the wash buffer, 20 ml of wash buffer concentrate was diluted into 480 ml of distilled water. To produce a stock solution of 2,000 pg/ml, the provided factor-standard was reconstituted with 1 ml of calibrator diluent and incubated for 15 min under gentle agitation. Following incubation, a standard dilution series was prepared with seven stages (2,000, 1,000, 500, 250, 125, 62.5 and 31.2 pg/ml). At the beginning of the test, 100 μl of assay diluent was added to each well of the mouse anti-human monoclonal capture antibody-coated microplate (IL-4, IL-6, EGFR, osteopontin, PDGF, G-CSF or VEGF)(R&D Systems). The first seven wells were filled with 100 μl of each standard dilution, and all other wells were filled with 100 μl of defrosted patient or control plasma samples. After a 2-h incubation, the wells were washed with wash buffer to remove unbound material. Subsequently, each well was filled with 200 μl of horseradish peroxidase-linked polyclonal goat anti-human detection antibody (IL-4, IL-6, EGFR, osteopontin, PDGF, G-CSF or VEGF)solution (factor conjugate; R&D Systems). The detection antibody bound another epitope of the antigen than the capture antibody, and as a result a sandwich of antibody-antigen-antibody emerged. After another 2-h incubation and washing, the wells were filled with the color substrate solution (stabilized horseradish peroxidase and tetramethylbenzidine; R&D Systems) and incubated for 25 min. The resulting color change in each well indicated the amount of antigen (factor) detected in the plasma. To terminate the enzymatic reaction, 50 μl of stop solution (2-N-sulfuric acid; R&D Systems) was added to each well. In the final step, the exact quantity of antigen was detected by a microplate reader (MRX-Reader; Dynatech Laboratories, Denkendorf, Germany) set at 450 nm.

### Statistical analysis

To calculate alterations in the pre- and post-therapeutic serum levels, the Wilcoxon signed-rank test for dependent samples was performed. For comparison of patients and controls, the t-test was used for normally distributed markers (Osteopontin, PDGF, EGFR, IL-4 and G-CSF). The levels of VEGF and IL-6 were not normally distributed. Consequently, both markers were analyzed with the non-parametric Mann-Whitney U-test. The statistical evaluation was conducted in cooperation with Dr C Weiss (Department of Medical Statistics, Biomathematics and Information Processing, Mannheim University Hospital, Mannheim, Germany). P<0.05 was considered to indicate a statistically significant difference.

## Results

### IL-4

**Figure 1:**
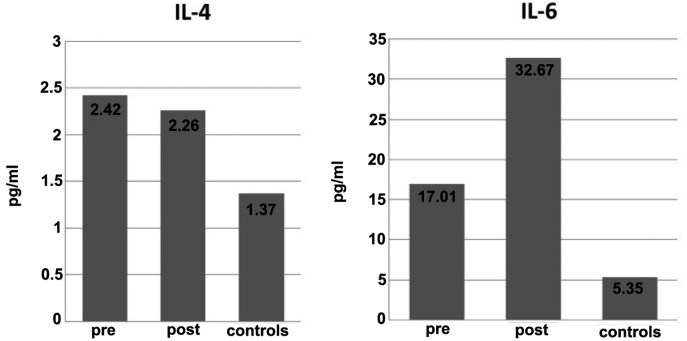
Mean IL-4 and IL-6 levels (pre- and post-therapeutic) in head and neck squamous cell carcinoma patients compared with healthy controls. IL, interleukin.

IL-4 exhibited the lowest values of all measured factors for patients and controls. The mean pre-therapeutic value of the patient group was 2.42±0.81 pg/ml, while the mean value of the control group was 1.37±0.63 pg/ml. Significant differences were shown between the control and patient groups (P=0.0001), with considerably higher IL-4 levels in the patient group. Following radiochemotherapy, the patient group showed a decline of 0.16±0.90 pg/ml in serum concentration (Table I and Fig. 1). Considering possible therapy-induced changes, the Wilcoxon signed-rank test revealed P=0.8500. Thus, a significance for therapy-associated IL-4 alterations was not shown, but a trend towards decreased levels was found.

### IL-6

Consistent with the results for IL-4, a significant increase of IL-6 levels in patients, P=0.0001, was found when comparing the IL-6 serum levels of patients and controls. The mean serum levels in the patient group (17.01±26.62 pg/ml) were three times those of the control group (5.35±17.89 pg/ml). A significant therapy-associated alteration of IL-6 (P=0.0300) was also shown in the patient group. The patients showed significantly elevated IL-6 serum levels (Table I and Fig. 1) following radiochemotherapy, with an average increase of 15.66±46.50 pg/ml.

### Osteopontin

**Figure 2:**
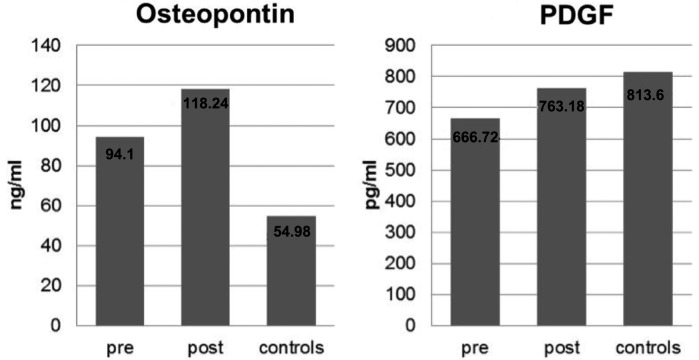
Mean osteopontin and PDGF levels (pre- and post-therapeutic) in head and neck squamous cell carcinoma patients compared with healthy controls. PDGF, platelet-derived growth factor.

The osteopontin levels in the patient and control samples showed distinct differences. The mean pre-therapeutic value of the patient group was 94.10±38.96 ng/ml, while the mean value of the control group was 54.98±20.97 ng/ml, and a statistical comparison of the groups revealed significantly higher osteopontin levels in patients (P=0.0003). Following radiochemotherapy, the patients showed a discrete but not significant increase of osteopontin levels (24.13±46.54 ng/ml; P=0.0600) (Table I and Fig. 2).

### PDGF

The levels of PDGF for the patient group were heterogeneous and unevenly distributed, with a mean value of 666.72±789.74 pg/ml. The mean value of the control group was 813.60±819.68 pg/ml. Statistical comparison of the groups showed P=0.4300. Following therapy, a mean increase of 96.46±480.68 pg/ml was measured in the patient group (Table I and Fig. 2). The P-value for the comparison of pre- and post-therapeutic levels was not significant (P=0.2600).

### VEGF

**Figure 3:**
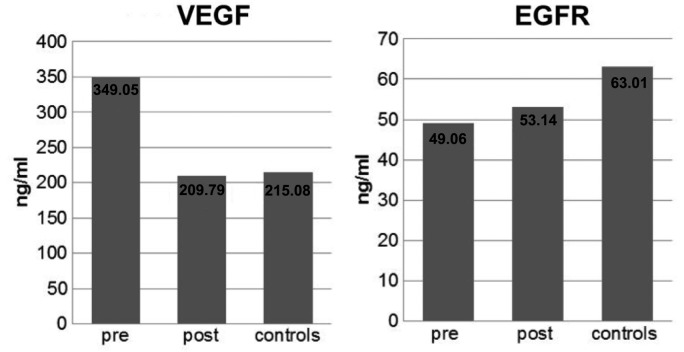
Mean VEGF and EGFR levels (pre- and post-therapeutic) in head and neck squamous cell carcinoma patients compared with healthy controls. VEGF, vascular endothelial growth factor; EFGR, epidermal growth factor receptor.

The results were inhomogeneous, with certain patients showing marked increases ≤444 pg/ml and others showing declines of 915 pg/ml following treatment. No reproducible tendency could be detected. As shown in Table I and Fig. 3, the mean concentration of VEGF decreased from 349.05±393.39 to 209.79±261.79 pg/ml following treatment. A statistically significant result was not exhibited for the statistical comparison of VEGF concentrations in patient serum prior and subsequent to treatment (P=0.3100). Furthermore, the comparison of controls and tumor patients was not statistically significant (P=0.9100). Consequently, a statistically significant result could not be stated for either the comparison of VEGF levels prior and subsequent to multimodal treatment or for the comparison of the levels in healthy controls and patients with HNSCC.

### EGFR

A homogenous distribution was found for the EGFR concentration in the tumor patients and control groups. The mean value of the control group (63.01±12.62 ng/ml) was significantly higher than the mean pre-therapeutic value of the patient group (49.06±15.58 ng/ml). Using the t-test for comparison of patients and controls, P=0.0005. In terms of the therapeutic process, the patient group presented with a mean difference of 1.90±18.86 ng/ml (data shown in Table I and Fig. 3). Statistical analysis of pre- and post-therapeutic results showed that P=0.4300 for therapy-induced concentration changes. Thus, no significant changes in EGFR were observed during therapy.

### G-CSF

**Figure 4:**
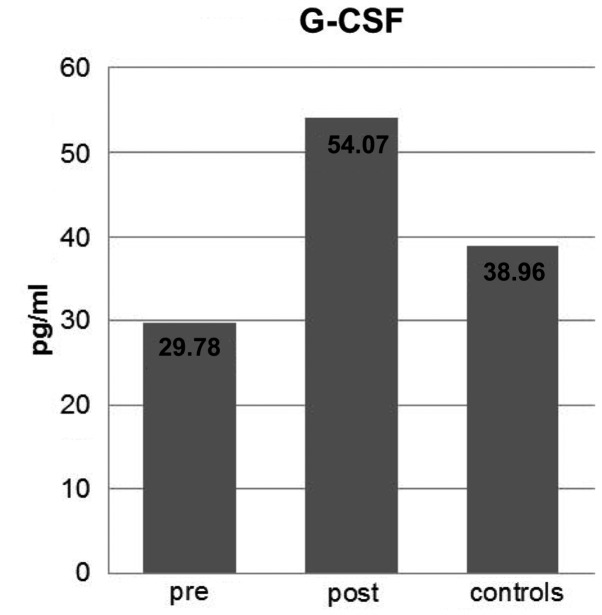
Mean G-CSF levels (pre- and post-therapeutic) in head and neck squamous cell carcinoma patients compared with healthy controls. G-CSF, granulocyte-colony stimulating factor.

The mean G-CSF concentration values for the patient (pre-therapy) and control groups were 29.79±10.83 and 38.96±51.27 pg/ml, respectively. However, no significant difference was identified between these two groups in terms of G-CSF concentration (P=0.9100). Following therapy, an increase of 24.29±81.40 pg/ml was measured in the patient group. The P-value for therapy-associated changes was 0.0600. Neither the differences between the pre- and post-therapy levels nor the comparison with the control group were significant (data shown in Table I and Fig. 4).

### Logistic regression

**Figure 5:**
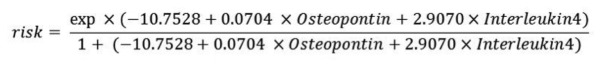
Formula for logistic regression.

For multivariate analysis, logistic regression was performed with osteopontin (P=0.0003) and IL-4 (P=0.0001) to compare patients and controls (Table I). Using these results, a formula was generated in which the osteopontin and IL-4 levels of a random patient could be calculated (Fig. 5). With this formula, an individual risk figure (from 0=low risk to 1=high risk) for the emergence of HNSCC can be created, including a corresponding Youden index (sensitivity + specificity − 1), which is applied as a marker for the quality of each test. The Youden index may be a value between −1 and +1, it is reasonable to apply a diagnostic test when the value is between 0 and +1. The closer the Youden index is to +1, the higher the diagnostic quality of a test. Thus, the higher the Youden index of a patient, the higher the likelihood for developing HNSCC depending on the individual serum levels of the two combined markers (osteopontin and IL-4). The higher the Youden index, the more reliable the generated risk figure (34).

**Figure 6:**
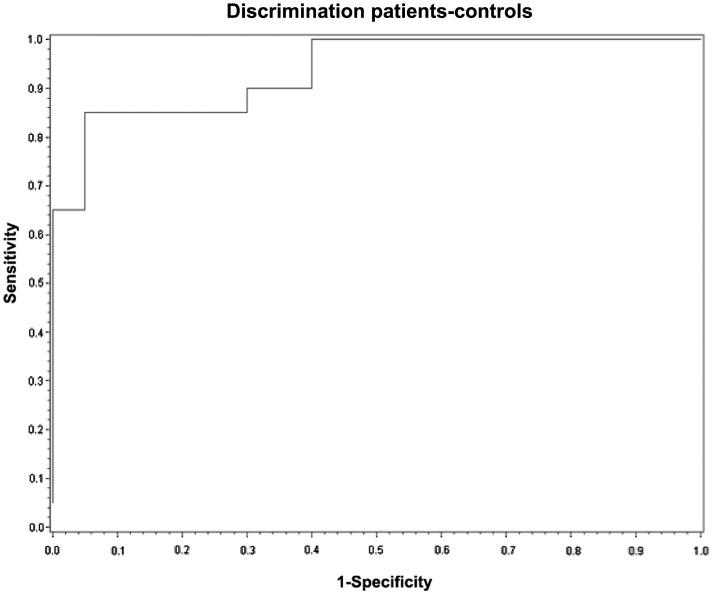
Receiver operating characteristic curve.

The results of the formula in Fig. 5 were used to create a receiver operating characteristic (ROC) curve in which sensitivity and 1-specificity were opposed (see Fig. 6). For each risk figure and corresponding Youden index, the curve shows the association between sensitivity and specificity and could aid in the diagnosis for each patient.

## Discussion

The present pilot study was performed to assess the validity of seven serological factors as biomarkers for malignant tumors of the head and neck. Furthermore, the study sought to investigate whether there are significant serum concentration differences of the analyzed factors between patients with HNSCC pre-therapeutic and healthy controls, and whether these markers are valid to indicate a neoplastic process of the head and neck as a screening instrument in primary diagnostic algorithms. Until now, tumor size and location, lymph node invasion, extracapsular spread and metastatic disease define the individual tumor prognosis without taking into account the heterogeneous tumor biology of the tumor entity (12). As another aim of the study, whether concomitant radiochemotherapy with cisplatin or carboplatin and 5-FU induces significant alterations of the serological levels of the seven surrogate markers compared with pre-therapeutic expression levels was examined.

Although there was no significant association with clinical and pathological parameters, two independent studies showed that patients with HNSCC present with higher levels of IL-4 compared with healthy controls (15,17). Klein (35) contradicted the study by Mojtahedi *et al* (17) and stated that IL-4 is not suitable for use as an HNSCC-screening marker. Therefore, the results concerning IL-4 are inconclusive. The results of the present study showed a significant tumor-associated increase of IL-4 when comparing patients with HNSCC and controls (P=0.0001), but unlike Mojtahedi *et al* (17), a significant decrease of IL-4 post-therapeutic (P=0.8500) was not found. Therefore, IL-4 appeared to be able to indicate a neoplastic process but was insufficient for monitoring the therapy response.

Wang *et al* (24) showed that patients with HNSCC present with increased IL-6 and IL-6 receptor levels compared with a healthy control group. Similarly, the present study documented an association between IL-6 levels, tumor size and histological grading (24). To identify IL-6 as a potential biomarker for HNSCC, Sato *et al* (36) proposed post-therapeutic saliva analysis for early detection of relapses. The results of the present study are consistent with the findings of Wang *et al* (24). IL-6 was significantly elevated in the patient serum (P=0.0001). However, a significant increase of IL-6 levels was also detected following therapy (P=0.0300). Therefore, IL-6 appears to be a suitable serological biomarker for malignant tumors of the head and neck. Clearly, therapy response cannot be indicated by IL-6 as the expression levels do not decrease following therapy.

Both Snitcovsky *et al* (37) and Weber *et al* (26) reported a significant correlation between the serological osteopontin concentration and tumor stage (26,37). Contrary to this, Lim *et al* (27) could not verify a correlation between elevated osteopontin levels in patients with HNSCC and a decreased overall survival rate or reduced therapy response (27). To a certain extent, the results of the present study confirmed the findings of Snitcovsky *et al* (37) who postulated greater levels of osteopontin in patients with advanced tumor stage. A significantly higher expression level was shown in the patient group compared with the chemotherapy-naive control group. During therapy, the patients in the study by Snitcovsky *et al* (37) presented with a mean decline of 14.5 ng/ml. By contrast, the patients in the present study showed an increase of 24.14±45.36 ng/ml following therapy (P=0.0600). These results found osteopontin to be potentially applicable for clinical use as a marker for tumor screening. However, osteopontin appeared to be unsuitable for use as a therapy response marker, as the results showed no significant changes in serum levels following radiochemotherapy with curative intent.

According to a study by Thariat *et al* (38), an overexpression of EGFR is detectable in 90% of all HNSCC cases and is associated with a poor overall survival rate. Regarding therapy-induced EGFR changes in patients, Bergler and Bier (39) recorded a 30% decline in therapy response among patients receiving platinum-based chemotherapy. On the contrary, the control group in the present study showed higher expression levels of EGFR compared with the patient group. However, a pathological overexpression of EGFR in oncologic patients could not be confirmed and, in fact, the opposite was true. Furthermore, a decrease of EGFR or any other significant therapy-associated changes was not found following treatment (P=0.4300). Therefore, EGFR cannot be recommended for use as either a biomarker or a screening parameter. This is in contrast to the findings of Riedel *et al* (40) who reported a downregulation of VEGF and endothelial cell migration following EGFR-targeted therapy.

In a multitude of neoplasmas, including breast, lung and head and neck cancer, an overexpression of VEGF has been detected previously (8). Various studies have been published on the correlation between tumor-node-metastasis (TNM) staging and VEGF levels. A study by Boonkitticharoen *et al* (9) showed a significant correlation between TNM staging and VEGF levels, however, a study by Riedel (11) did not. The results of the present study did not show a significant difference in the VEGF serum levels of patients prior or subsequent to treatment (P=0.3100). Nor was a difference between patients and controls detected (P=0.9100). Based on these results, VEGF serum levels cannot be recommended as a prognostic parameter.

Palmer *et al* (13) showed that patients with HNSCC have significantly higher PDGF levels compared with a control group, however, similar results were not stated in the present study. In the patient and control groups, the PDGF levels were heterogeneous and unevenly distributed. Therefore, significant results were not shown for either the comparison of patients and controls (P=0.4300) or for therapy-associated changes of the patient group (P=0.2600). Considerable discrepancies were found between the mean values of patients and controls in the present study and the study by Palmer *et al* (13). The results of the present study revealed mean values of 813.60±809.36 pg/ml in controls and 666.72±769.74 pg/ml in patients, while Palmer *et al* revealed mean values of 1,708.52 pg/ml in controls and 5,945.28 pg/ml in patients. Both studies used ELISA for the detection of PDGF. However, the results of the study by Palmer *et al* exceed the present study by nine-fold, which is a remarkable difference. Based on the present study results, it can be concluded that PDGF is not suitable as a biomarker for HNSCC or for the analysis of therapy response.

In HNSCC, G-CSF stimulates proliferation and migration of tumor and inflammatory cells. In contrast to G-CSF-negative tumors, G-CSF-positive tumors are distinctively invasive of the bone and cartilage tissues (31). Besides HNSCC, lung, uterus and hepatocellular carcinomas present with elevated G-CSF levels and are associated with a poor outcome (41–43). The present study revealed approximately the same mean values in the patient and control groups. There was no significant difference in the comparison of the groups (P=0.9100) and elevated serum levels following therapy (P=0.0600) were not significant. Based on these results, G-CSF is not suitable as a screening marker or as a marker for therapy-induced alterations of the serological marker signature.

In conclusion, the present pilot study revealed a significant correlation between three serological markers (osteopontin, IL-4 and IL-6) and a histopathologically confirmed neoplasm of the head and neck. The comparison between serum samples of tumor patients and the control group showed significantly elevated serum levels of osteopontin, IL-4 and IL-6. Therefore, these markers could be a suitable tool in the primary diagnostic algorithm of a head and neck tumor (screening instrument). Only IL-6 showed a significant difference (an increase) in the expression levels post-therapeutically. Thus, none of the markers may be used as an indicator of treatment response, since a reduction of the elevated expression levels would be expected following sufficient therapy. Taking into account the clinically observed post-therapeutic local and regional tumor control of the tumor patient collective, the present study failed to identify a serological multi-marker strategy as sufficient to monitor treatment success and predict the individual prognosis of tumor disease.

Logistic regression facilitates the calculation of the individual risk for HNSCC using osteopontin and IL-4. By using the results of the present study with the formula (Fig. 5), a ROC curve was created in which sensitivity and 1-specificity were opposed (see Fig. 6). For each risk figure and corresponding Youden index, the curve shows the association between sensitivity and specificity. This can be observed as a quantification of test quality for the screening of HNSCC. The suitability of this procedure for clinical use requires investigation in clinical trials. Based on these results, a serological multi-marker strategy for screening diagnosis and follow-up requires further evaluation. IL-4, IL-6 and osteopontin appeared to be suitable as screening parameters in the diagnosis of HNSCC. However, none of these parameters were sufficient for indicating the therapy response as the possible markers for screening and diagnosis that showed elevated levels in tumor patients did not reveal a consistent decrease following sufficient therapy.

## Figures and Tables

**Table I:** Serum levels of patients with HNSCC and the control group prior and subsequent to therapy.

Marker	Patients^a^	Controls^a^	Difference pre-post treatment patients^a^	P-value patients-controls	P-value patients pre-post treatment
Osteopontin (ng/ml)	94.10±38.96	54.98±20.97	24.13±46.54	0.0003^b^	0.06
PDGF (pg/ml)	666.72±789.74	813.60±819.68	96.46±480.68	0.4300	0.26
VEGF (pg/ml)	349.05±403.61	215.08±208.39	−139.26±405.56	0.9100	0.31
EGFR (ng/ml)	49.06±15.99	63.01±12.78	1.90±18.86	0.0005^b^	0.43
IL-4 (pg/ml)	2.42±0.81	1.37±0.63	−0.16±0.90	0.0001^b^	0.85
IL-6 (pg/ml)	17.01±25.62	5.35±17.89	15.66±46.50	0.0001^b^	0.03^b^
G-CSF (pg/ml)	29.79±10.83	38.96±51.27	24.29±81.40	0.9100	0.06

a Results are presented as the mean ± standard deviation;

b statistically significant.

HNSCC, head and neck squamous cell carcinoma; PDGF, platelet-derived growth factor; VEGF, vascular endothelial growth factor; EFGR, epidermal growth factor receptor; IL, interleukin, G-CSF, granulocyte-colony stimulating factor.

## Abbreviations

5-FU: 5-fluorouracil
EGF: epidermal growth factor
EGFR: EGF receptor
ELISA: enzyme-linked immunosorbent assay
G-CSF: granulocyte-colony stimulating factor
HNSCC: head and neck squamous cell carcinoma
IL: interleukin
PDGF: platelet-derived growth factor
ROC: receiver operating characteristic
VEGF: vascular endothelial growth factor
